# Supplementary material for: The assessment of physical risk taking: Preliminary construct validation of a new behavioral measure
Source: PLoS One. 2021 Oct 28;16(10):e0258826. doi: 10.1371/journal.pone.0258826 (PMC8553120; doi:10.1371/journal.pone.0258826)
Supplement: S1 Table — Numbers in interior cells indicate the number of trials containing those specific APRT conditions. (DOCX) [file pone.0258826.s001.docx]

| APRT Condition | 1 | 2 | 3 | 4 | 5 | 6 | 7 | 8 | 9 | 10 | 11 | 12 |
| --- | --- | --- | --- | --- | --- | --- | --- | --- | --- | --- | --- | --- |
| 1. Picture Type (Animal) | 16 | 0 | 0 | 0 | 8 | 8 | 8 | 8 | 8 | 8 | 8 | 8 |
| 1. Picture Type (Cliff) |  | 16 | 0 | 0 | 8 | 8 | 8 | 8 | 8 | 8 | 8 | 8 |
| 1. Picture Type (Disaster) |  |  | 16 | 0 | 8 | 8 | 8 | 8 | 8 | 8 | 8 | 8 |
| 1. Picture Type (Hero) |  |  |  | 16 | 8 | 8 | 8 | 8 | 8 | 8 | 8 | 8 |
| 1. Injury Magnitude (High) |  |  |  |  | 32 | 0 | 16 | 16 | 16 | 16 | 16 | 16 |
| 1. Injury Magnitude (Low) |  |  |  |  |  | 32 | 16 | 16 | 16 | 16 | 16 | 16 |
| 1. Reward Magnitude (High) |  |  |  |  |  |  | 32 | 0 | 16 | 16 | 16 | 16 |
| 1. Reward Magnitude (low) |  |  |  |  |  |  |  | 32 | 16 | 16 | 16 | 16 |
| 1. Injury Probability (High) |  |  |  |  |  |  |  |  | 32 | 0 | 16 | 16 |
| 1. Injury Probability (Low) |  |  |  |  |  |  |  |  |  | 32 | 16 | 16 |
| 1. Reward Probability (High) |  |  |  |  |  |  |  |  |  |  | 32 | 0 |
| 1. Reward Probability (Low) |  |  |  |  |  |  |  |  |  |  |  | 32 |
