# Supplementary material for: The assessment of physical risk taking: Preliminary construct validation of a new behavioral measure
Source: PLoS One. 2021 Oct 28;16(10):e0258826. doi: 10.1371/journal.pone.0258826 (PMC8553120; doi:10.1371/journal.pone.0258826)
Supplement: S2 Table — (DOCX) [file pone.0258826.s002.docx]

|  | *N* | *M* | *SD* | Skewness | Kurtosis | α |
| --- | --- | --- | --- | --- | --- | --- |
| **Points** |  |  |  |  |  |  |
| Picture Type (Hero – Disaster) | 246 | 61.86 | 186.22 | .78 | 2.76 | .31 |
| Picture Type (Cliff – Animal) | 246 | 22.72 | 213.88 | .09 | 3.41 | .22 |
| Injury Magnitude | 247 | -127.66 | 170.91 | -.14 | 1.29 | .76 |
| Reward Magnitude | 246 | 340.87 | 272.58 | .72 | -.69 | .89 |
| Injury Probability | 247 | 196.60 | 201.48 | .84 | -.46 | .82 |
| Reward Probability | 247 | 99.15 | 104.67 | 1.23 | 1.25 | .42 |
| **Go Presses** |  |  |  |  |  |  |
| Picture Type (Hero – Disaster) | 246 | .89 | 2.85 | 1.09 | 3.72 | .44 |
| Picture Type (Cliff – Animal) | 246 | .34 | 3.15 | 1.05 | 4.95 | .43 |
| Injury Magnitude | 247 | -2.86 | 3.85 | -1.54 | 1.62 | .87 |
| Reward Magnitude | 246 | -.38 | 1.99 | -.16 | 2.21 | .42 |
| Injury Probability | 247 | 4.15 | 4.27 | .98 | -.06 | .88 |
| Reward Probability | 247 | -.20 | 1.58 | -.04 | 3.25 | .28 |
| **Remaining Health** |  |  |  |  |  |  |
| Picture Type (Hero – Disaster) | 246 | -4.57 | 12.61 | -1.08 | 3.06 | .44 |
| Picture Type (Cliff – Animal) | 246 | -3.98 | 12.08 | -.86 | 3.28 | .47 |
| Injury Magnitude | 247 | -16.67 | 11.66 | -.43 | -.13 | .72 |
| Reward Magnitude | 246 | 1.12 | 8.21 | -.10 | 1.73 | .34 |
| Injury Probability | 247 | 8.41 | 8.80 | .29 | .44 | .48 |
| Reward Probability | 247 | .62 | 6.88 | .02 | .57 | .24 |
| **Injuries** |  |  |  |  |  |  |
| Picture Type (Hero – Disaster) | 246 | .12 | .38 | 1.14 | 4.04 | .48 |
| Picture Type (Cliff – Animal) | 246 | .08 | .40 | .85 | 4.00 | .40 |
| Injury Magnitude | 247 | -.44 | .58 | -1.36 | .88 | .91 |
| Reward Magnitude | 246 | -.05 | .23 | -.10 | 1.64 | .34 |
| Injury Probability | 247 | -.26 | .27 | -.61 | .75 | .59 |
| Reward Probability | 247 | -.02 | .18 | -.21 | .30 | .21 |
