# Supplementary material for: The assessment of physical risk taking: Preliminary construct validation of a new behavioral measure
Source: PLoS One. 2021 Oct 28;16(10):e0258826. doi: 10.1371/journal.pone.0258826 (PMC8553120; doi:10.1371/journal.pone.0258826)
Supplement: S3 Table — (DOCX) [file pone.0258826.s003.docx]

|  | *N* | *M* | *SD* | Skewness | Kurtosis | α |
| --- | --- | --- | --- | --- | --- | --- |
| **UPPS-P** |  |  |  |  |  |  |
| Negative Urgency | 223 | 28.79 | 7.31 | 0.21 | -0.32 | .85 |
| Positive Urgency | 223 | 28.27 | 8.90 | 0.56 | 0.11 | .92 |
| Lack of Premeditation | 223 | 19.44 | 5.13 | 0.51 | 0.13 | .82 |
| Perseverance | 223 | 19.48 | 4.86 | 0.43 | -0.19 | .80 |
| Sensation Seeking | 223 | 33.61 | 7.43 | -0.28 | -0.66 | .84 |
| **SSS** |  |  |  |  |  |  |
| Disinhibition | 225 | 4.52 | 2.46 | 0.15 | -0.79 | .68 |
| Boredom Susceptibility | 225 | 2.60 | 1.78 | 0.51 | -0.22 | .57 |
| Thrill Seeking | 225 | 5.36 | 3.01 | -0.04 | -1.10 | .82 |
| Experience Seeking | 225 | 5.06 | 1.97 | 0.11 | -0.48 | .52 |
| **SHART** |  |  |  |  |  |  |
| Sex (Perception) | 224 | 46.38 | 8.94 | -0.47 | -0.52 | .89 |
| Driving (Perception) | 224 | 41.05 | 6.81 | -0.03 | -0.43 | .86 |
| Sex (Taking) | 223 | 16.54 | 4.28 | 1.78 | 5.41 | .78 |
| Driving (Taking) | 224 | 29.63 | 10.10 | 0.24 | -0.74 | .90 |
| **DOSPERT** |  |  |  |  |  |  |
| Ethics (Taking) | 226 | 14.25 | 5.27 | 0.65 | 0.20 | .52 |
| Financial (Taking) | 226 | 14.94 | 5.86 | 0.82 | 1.39 | .70 |
| Health & Safety (Taking) | 226 | 18.29 | 7.04 | 0.43 | 0.10 | .60 |
| Recreational (Taking) | 226 | 22.36 | 9.52 | 0.16 | -0.94 | .84 |
| Social (Taking) | 226 | 27.93 | 5.80 | -0.14 | 0.00 | .55 |
| Ethics (Perception) | 226 | 29.98 | 5.39 | -0.45 | 0.75 | .56 |
| Financial (Perception) | 226 | 30.09 | 6.67 | -0.64 | 0.76 | .74 |
| Health & Safety (Perception) | 226 | 31.93 | 6.01 | -0.76 | 1.11 | .69 |
| Recreational (Perception) | 226 | 27.98 | 6.46 | -0.59 | 0.78 | .72 |
| Social (Perception) | 226 | 19.69 | 5.50 | 0.31 | 0.11 | .60 |
| **BHRQ** |  |  |  |  |  |  |
| Childhood Aggression | 223 | 13.36 | 2.56 | 1.83 | 4.95 | .68 |
| Childhood Non-aggression | 222 | 28.90 | 6.04 | 1.40 | 2.12 | .79 |
| Adult Aggression | 222 | 15.42 | 3.44 | 1.12 | 1.51 | .69 |
| Adult Non-aggression | 222 | 26.67 | 5.15 | 2.01 | 5.61 | .81 |
| **SDAST** | 222 | 1.44 | 2.42 | 3.53 | 16.91 | .76 |
| **ADS** | 218 | 38.38 | 4.01 | 1.76 | 4.06 | .86 |
| **CRS** | 219 | 13.39 | 4.68 | 0.27 | -0.84 | .96 |
