# Supplementary material for: The assessment of physical risk taking: Preliminary construct validation of a new behavioral measure
Source: PLoS One. 2021 Oct 28;16(10):e0258826. doi: 10.1371/journal.pone.0258826 (PMC8553120; doi:10.1371/journal.pone.0258826)
Supplement: S4 Table — *padj < .05. ** padj < .01. *** padj < .001. (DOCX) [file pone.0258826.s004.docx]

| APRT SCORE (NO DELAY/DELAY) | | | | | | | |
| --- | --- | --- | --- | --- | --- | --- | --- |
| Self-Report Scale | Injury Magnitude | Injury Probability | Animal-Cliff | Hero-Disaster | Reward Magnitude | Reward Probability | APRT total |
| SSS Thrill-Seeking | -.216/-.158 | .020/-.158 | -.093/-.185 | .019/-.025 | .195/.025 | .058/.000 | -.312^***^/-.199 |
| DOSPERT Recreational Risk taking | -.256^*^/-.180 | -.056/-.177 | -.010/-.095 | .089/.065 | .119/.123 | .115/-.072 | -.323^***^/-.235 |
| UPPS-P Sensation Seeking | -.126/-.158 | -.007/-.064 | -.066/-.146 | .102/-.028 | .093/.042 | .151/-.058 | -.303^**^/-.183 |
| SHART Driving Risk taking | -.221/-.025 | -.098/.069 | -.015/-.079 | .211/.090 | .130/.038 | .042/-.005 | -.219/-.148 |
| DOSPERT Social Risk taking | -.137/-.074 | -.170/.025 | .035/-.097 | .058/.033 | -.093/-.063 | .021/.095 | -.162/-.21 |
| DOSPERT Health/Safety Risk taking | -.208/.057 | -.069/.123 | -.067/-.139 | .098/.103 | .099/-.105 | .077/-.185 | -.093/-.137 |
| DOSPERT Ethics Risk taking | -.159/-.004 | -.127/.027 | .060/-.068 | .169/.183 | -.019/-.013 | .233/-.052 | -.145/-.091 |
| SHART Sexual Risk taking | -.099/.086 | -.076/.239 | -.149/.007 | .002/.060 | .119/-.064 | .020/-.028 | -.027/-.020 |
| DOSPERT Financial Risk taking | -.087/.134 | -.154/.164 | -.086/-.119 | -.077/-.102 | .060/-.045 | .203/.117 | -.055/.048 |
| DOSPERT Recreational Risk-Perception | .030/.070 | -.037/.071 | -.117/.189 | .007/.011 | -.149/.063 | -.133/.040 | .216/.140 |
| SHART Driving Risk-Perception | .106/.063 | .126/-.008 | -.152/-.020 | -.149/-.050 | -.052/.045 | .062/-.050 | .182/.171 |
| BHRQ Adulthood Aggressive | -.062/.020 | -.137/.097 | .026/.015 | .231/.126 | .023/-.042 | .199/-.097 | -.127/-.103 |
| SSS Boredom Susceptibility | -.176/-.036 | -.187/-.039 | -.050/.019 | .194/.062 | -.104/.002 | .167/.063 | -.251^*^/-.077 |
| SHART Sexual Risk-Perception | .147/.009 | -.013/-.097 | -.036/-.005 | -.086/-.073 | -.027/.173 | .004/.001 | .221/.026 |
| DOSPERT Ethical Risk-Perception | .178/-.087 | .026/-.070 | -.119/.146 | -.242/-.023 | -.080/.033 | -.158/.135 | .202/.017 |
| BHRQ Childhood Aggressive | -.110/-.004 | -.180/.117 | -.033/.057 | .214/.256^*^ | .067/.083 | .093/-.133 | .007/-.136 |
| DOSPERT Health/Safety Risk-Perception | .046/-.100 | -.010/-.110 | -.162/.165 | -.134/-.064 | -.035/.048 | -.059/.045 | .118/.057 |
| ADS Total | -.238/.194 | -.077/.214 | -.086-/.056 | .120/-.008 | .145/-.088 | -.002/-.050 | -.152/.009 |
| BHRQ Childhood Non-aggressive | -.075/-.090 | -.055/.100 | .046/.060 | .137/.187 | -.003/.204 | .046/-.131 | -.006/-.125 |
| DOSPERT Financial Risk-Perception | .005/.117 | -.038/.063 | .047/.157 | -.004/-.103 | -.098/.006 | -.110/-.163 | .170/.088 |
| SDAST Total | -.183/-.034 | -.181/.166 | .009/.076 | -.078/.037 | .194^*^/-.064 | .254^*^/-.004 | -.018/-.111 |
| SSS Experience Seeking | -.258^*^/.040 | -.101/.092 | -.145/-.104 | -.047/.028 | .066/-.064 | .086/-.010 | -.051/.018 |
| UPPS-P Negative Urgency | -.130/-.033 | -.108/.015 | -.176/.013 | .050/.101 | .003/.112 | .091/-.095 | -.027/-.035 |
| CRS Total | -.034/.122 | -.104/.019 | -.058/-.067 | -.090/.047 | .139/.180 | -.009/-.069 | .023/-.009 |
| UPPS-P Perseverance | -.004/-.007 | -.157/.057 | -.041/.025 | -.097/.050 | .017/.010 | .072/.138 | .109/.100 |
| UPPS-P Positive Urgency | -.262^*^-.068 | -.076/-.027 | -.040/-.001 | .113/.063 | .045/.062 | .144/-.063 | -.065/.060 |
| SSS Disinhibition | -.173/.151 | -.130/.100 | -.102/-.103 | .170/-.050 | .129/-.132 | .100/-.015 | -.080/.079 |
| BHRQ Adulthood Non-aggressive | -.073/.118 | -.034/.281^*^ | -.016/.030 | .111/-.016 | -.038/-.023 | .084/-.035 | -.031/.027 |
| UPPS-P Lack of Premeditation | -.083/.127 | -.103/.186 | -.210^*^/-.115 | -.106/.131 | .068/-.087 | .110/.045 | -.029/.014 |
| DOSPERT Social Risk-Perception | .002/.021 | -.069/.024 | -.033/.042 | .126/-.080 | .058/.011 | -.153/.095 | .027/.014 |
