# Supplementary material for: The assessment of physical risk taking: Preliminary construct validation of a new behavioral measure
Source: PLoS One. 2021 Oct 28;16(10):e0258826. doi: 10.1371/journal.pone.0258826 (PMC8553120; doi:10.1371/journal.pone.0258826)
Supplement: S5 Table — *padj < .05. ** padj < .01. *** padj < .001. (DOCX) [file pone.0258826.s005.docx]

| APRT SCORE (NO DELAY/DELAY) | | | | | | | |
| --- | --- | --- | --- | --- | --- | --- | --- |
| Self-Report Scale | Injury Magnitude | Injury Probability | Animal-Cliff | Hero-Disaster | Reward Magnitude | Reward Probability | APRT total |
| SSS Thrill-Seeking | -.241/-.120 | .053/.181 | .104/.177 | -.031/.005 | -.150/-.068 | -.049/-.061 | .304^**^/.196 |
| DOSPERT Recreational Risk taking | -.225/-.145 | .135/.209 | -.017/.101 | -.104/-.071 | -.064/-.105 | -.097/.004 | .308^**^/.232 |
| UPPS-P Sensation Seeking | -.270^*^/-.109 | .101/.099 | .012/.167 | -.104/-.004 | -.058/-.045 | -.115/.047 | .305^**^/.177 |
| SHART Driving Risk taking | -.143/-.189 | .137/-.004 | .033/.045 | -.151/-.088 | -.067/-.015 | .010/.050 | .212/.167 |
| DOSPERT Social Risk taking | -.124/-.235 | .153/-.027 | -.058/.074 | .054/-.061 | .098/.128 | -.009/-.038 | .149/.224 |
| DOSPERT Health/Safety Risk taking | -.017/-.256^*^ | .102/-.114 | .034/.176 | -.054/-.118 | -.076/.050 | -.044/.200 | .084/.170 |
| DOSPERT Ethics Risk taking | -.097/-.126 | .154/-.037 | -.098/.098 | -.194/-.113 | -.045/.046 | -.197/.066 | .135/.109 |
| SHART Sexual Risk taking | .001/-.122 | .171/-.230 | .125/.018 | -.008/-.118 | -.140/.026 | -.050/.000 | .028/.052 |
| DOSPERT Financial Risk taking | .002/-.042 | .112/-.115 | .074/.087 | .053/.114 | -.063/.139 | -.219/-.041 | .045/-.022 |
| DOSPERT Recreational Risk-Perception | .218^*^/.129 | -.036/-.088 | .071/-.161 | .032/.001 | .057/.020 | .164/.025 | -.224/-.150 |
| SHART Driving Risk-Perception | .134/.189 | -.110/.011 | .109/-.012 | .125/.039 | .036/-.032 | -.080/.023 | -.182/-.183 |
| BHRQ Adulthood Aggressive | -.117/-.177 | .225/-.022 | -.021/.008 | -.195/-.123 | -.012/.014 | -.108/.126 | .140/.126 |
| SSS Boredom Susceptibility | -.175/-.033 | .255^*^/.024 | .040/-.024 | -.164/-.118 | .075/-.017 | -.150/-.141 | .249^*^/.076 |
| SHART Sexual Risk-Perception | .145/.039 | -.076/.117 | .034/.002 | .114/.097 | -.056/-.099 | .014/.040 | -.217/-.038 |
| DOSPERT Ethical Risk-Perception | .152/.110 | -.056/.088 | .114/-.172 | .196/.005 | .032/.005 | .124/-.082 | -.200/-.045 |
| BHRQ Childhood Aggressive | .068/-.209 | .201/-.061 | .008/.003 | -.199/-.229 | -.055/-.113 | -.044/.100 | -.015/.158 |
| DOSPERT Health/Safety Risk-Perception | .087/.182 | .000/.115 | .100/-.175 | .061/-.007 | -.024/.045 | .088/.007 | -.131/-.086 |
| ADS Total | -.060/-.173 | .151/-.161 | .060/.015 | -.122/-.077 | -.091/.043 | .017/.006 | .148/.030 |
| BHRQ Childhood Non-aggressive | .042/-.092 | .113/-.050 | -.047/-.077 | -.104/-.172 | -.021/-.226 | .001/.054 | .000/.128 |
| DOSPERT Financial Risk-Perception | .147/.018 | .025/-.078 | -.030/-.135 | .088/.079 | .137/.035 | .153/.150 | -.174/-.090 |
| SDAST Total | .081/-.140 | .209/-.123 | -.022/-.027 | .041/-.078 | -.200/.086 | -.253^*^/.002 | .016/.117 |
| SSS Experience Seeking | .066/-.013 | .162/-.049 | .122/.110 | .108/-.083 | -.059/.085 | -.036/-.001 | .041/-.010 |
| UPPS-P Negative Urgency | .054/-.044 | .137/.030 | .128/.035 | -.093/-.064 | -.040/-.045 | -.057/.122 | .014/.038 |
| CRS Total | .064/-.142 | .017/.051 | .101/-.014 | .072/-.008 | -.148/-.143 | -.003/.126 | -.036/.039 |
| UPPS-P Perseverance | .118/.147 | .191/-.088 | .079/-.113 | .005/.037 | -.069/.005 | -.117/-.154 | -.105/-.107 |
| UPPS-P Positive Urgency | .068/.013 | .076/.029 | .009/.010 | -.115/-.039 | -.061/-.062 | -.107/.051 | .036/-.044 |
| SSS Disinhibition | .001/-.015 | .236/-.059 | .015/.130 | -.145/-.068 | -.112/.122 | -.084/-.030 | .082/-.058 |
| BHRQ Adulthood Non-aggressive | -.009/-.086 | .085/-.225 | .026/-.021 | -.049/-.013 | .054/.007 | -.039/.062 | .033/-.004 |
| UPPS-P Lack of Premeditation | .022/-.115 | .112/-.163 | .209/.061 | .066/-.080 | -.108/.075 | -.039/-.055 | .022/.032 |
| DOSPERT Social Risk-Perception | .048/-.002 | .036/.044 | -.045/.022 | -.175/.092 | -.074/.065 | .195/.038 | -.036/-.009 |
