# Supplementary material for: The assessment of physical risk taking: Preliminary construct validation of a new behavioral measure
Source: PLoS One. 2021 Oct 28;16(10):e0258826. doi: 10.1371/journal.pone.0258826 (PMC8553120; doi:10.1371/journal.pone.0258826)
Supplement: Supplementary file 6 [file pone.0258826.s006.docx]

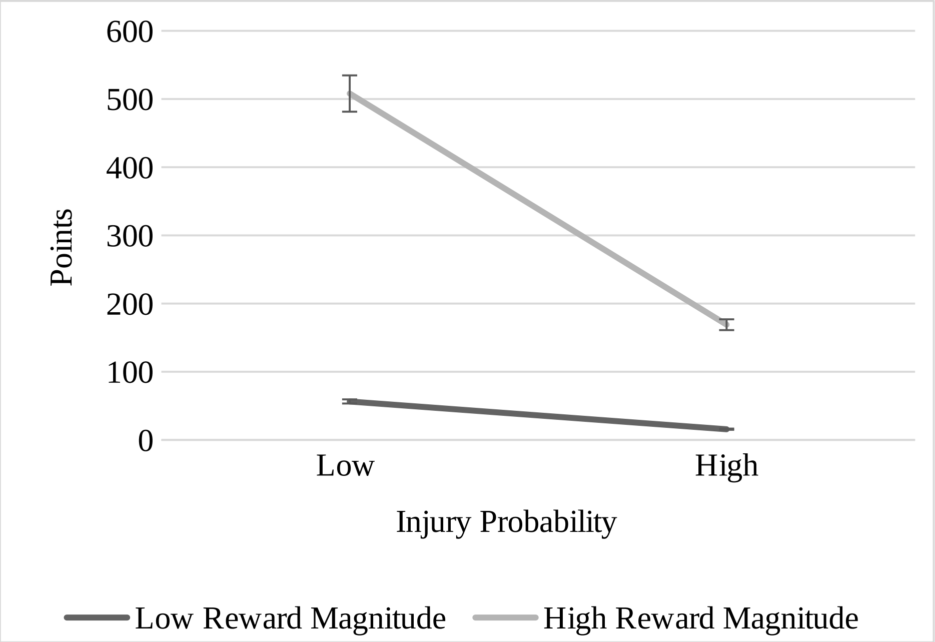

600
500
400
300
200
100
0
Points
Low
High
Injury Probability
Low Reward Magnitude
High Reward Magnitude
