# Supplementary material for: The assessment of physical risk taking: Preliminary construct validation of a new behavioral measure
Source: PLoS One. 2021 Oct 28;16(10):e0258826. doi: 10.1371/journal.pone.0258826 (PMC8553120; doi:10.1371/journal.pone.0258826)
Supplement: Supplementary file 7 [file pone.0258826.s007.docx]

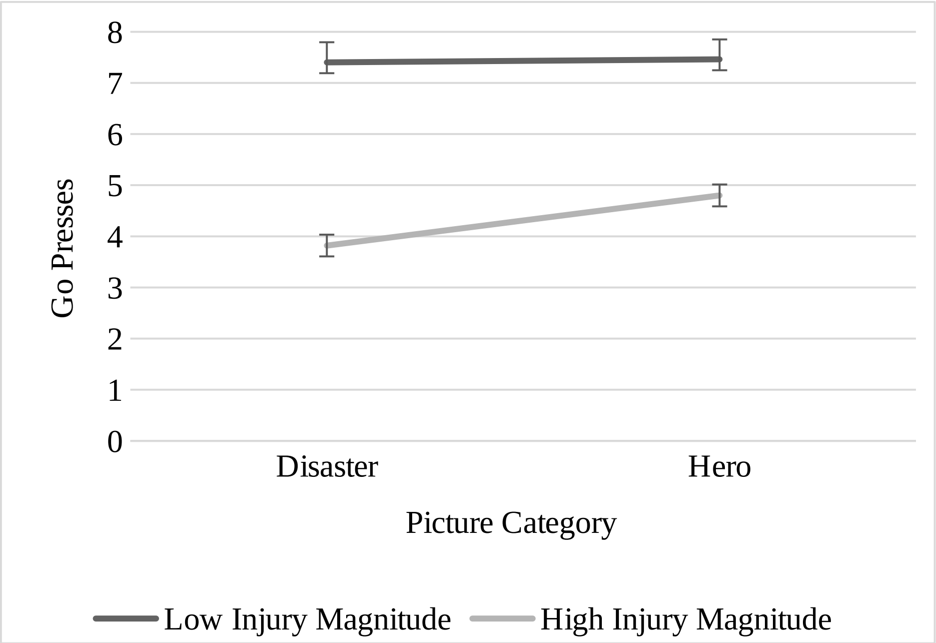
8
7
6
5
4
3
2
1
0
Go Presses
Disaster
Hero
Picture Category
Low Injury Magnitude
High Injury Magnitude
