# Supplementary material for: The assessment of physical risk taking: Preliminary construct validation of a new behavioral measure
Source: PLoS One. 2021 Oct 28;16(10):e0258826. doi: 10.1371/journal.pone.0258826 (PMC8553120; doi:10.1371/journal.pone.0258826)
Supplement: Attachment — Submitted filename: Response to Reviewers.docx [file pone.0258826.s009.docx]

May 07, 2021

Dear Dr. Konstantinidis,

We very much appreciate the opportunity to revise our previous manuscript on our new behavioral task, the Assessment of Physical Risk Taking. Doing so has allowed us to strengthen our arguments and expand our review of the literature, as well as update the title of our submission (see below). The manuscript and supplemental materials have been updated to reflect PLoS ONE formatting guidelines. Below, we detail our responses to both reviewer’s comments.

*Reviewer 1*

1. *This paper presents a novel behavioral task aimed at assessing "physical risk taking". The authors claim that one of the task's main goals is to disentangle the relative influences of the i) probability and ii) size of the reward, as well as the iii) probability and iv) size of the loss that may result from taking a risk. I agree with the authors that improved behavioral tasks of risk taking are much needed. Moreover, the empirical study has been conducted carefully, and I appreciate that the materials and data are openly accessible. That said, I have several conceptual questions and comments, and overall the central goal and contribution of the paper remained somewhat unclear to me: Is it primarily to develop a novel measure for "physical" risk taking (if so: why exactly physical risk taking)? Is it to disentangle the potential drivers of risk taking as mentioned above (if so: why was this done in a "novel" domain such as "physical risk taking" and not using one of the more established domains)? Is it to study "multi-dimensional" risk problems (i.e., as opposed to the typical gambles, where the probability of a reward is often the inverse of the probability of a loss)? Is it to further examine self-regulation and impulsivity as drivers of risk taking (which seems to be implied by the hot / cold versions of the task)? Or is it about a combination of all of the above? Taken together, all in all I am not (yet) entirely convinced that this task lives up to the expectations, and several issues need to be addressed in order to better clarify the exposition of this research. I hope that my comments and questions below will be helpful to this end.*

We appreciate this critique, as clarifying our goals is indeed critical to establishing the impact of this manuscript. As was suggested, our goals were a combination of several of the above, and it is our hope that our responses to each of the comments and questions below adequately address this concern. In particular, the representative design framework advanced in Steiner and Frey (2021) proved to be an integral conceptual framework that allowed us to better describe why photorealistic tasks that manipulate multiple factors of risk taking simultaneously (like APRT) was needed.

2. *The authors argue that "most definitions of risk focus exclusively on potential negative consequences". Although it is true that some disciplines adopt the view of risk taking as behavior with potentially harmful consequences, this general statement is not entirely correct. In economics (and economic psychology), for instance, risk is typically defined as "outcome variance" (see Schonberg et al., 2011) and hence "risk" also exist in gains-only settings.*

We greatly valued this point and have expanded the introduction (pp. 3-4) to review alternative definitions of risk beyond the original conceptualization we reviewed (i.e., exclusive focus on negative consequences). We cited Schonberg and colleagues (2011) as suggested, and we also mention definitions of risk in gain-only circumstances and definitions that involve both loss and gain as distinct factors.

3. *Relatedly, contrary to the portrayal in the introduction it is not the case that psychological theories of behavioral risk taking exclusively focus(ed) on the role of negative consequences. In fact, studies in the field of decision analysis (often using monetary gambles) have examined many risk-related phenomena purely in the gain domain (e.g., EU-theory). Consequently, the documentation of the "reflection effect" started to draw attention on differential effects of gains and losses, triggering the introduction of a reference point in prospect theory. To some extent, the relative importance of gains and losses has also been studied in the BART (which serves as an important starting point in the current paper), with respective models taking into account reward sensitivity, loss aversion, etc. (Wallsten et al., 2005).*

Continuing from the previous point, we have also expanded our review of current risk taking measures to include research on the differential effects of loss and gain on participant behavior on both BART and IGT task performance, including the Wallsten et al. (2005) models of BART performance (pp. 5-7).

4. *The authors write that "current behavioral task correlate with real-world risky behaviors broadly", but there is no reference to support this claim. In fact, accumulating empirical evidence indicates that behavioral tasks may lack external validity.*

We were extremely grateful to have this point highlighted. We have removed this statement from our introduction and instead note that the literature does indeed suggest many extant tasks likely lack adequate external validity (p. 4).

5. *Contrary to the authors' claim, many attempts have been made to decompose various tasks into basic behavioral components. As outlined above, this has been the case for the BART, but naturally also for the widespread monetary lotteries (i.e., the associated choice models often differentiate between the roles of gains / losses and the respective probabilities; cf. the fourfold pattern of risk). The same is true for the Iowa Gambling Task (cf. Yechiam, 2005).*

As mentioned in our response to the second point, we have expanded our literature review, which now includes the IGT models constructed by Yechiam (2005) on p. 7, paragraph 1.

6. *It is not quite true that the "injury probability condition is almost never considered in the BART" (p. 5; as a minor comment, it seemed a bit odd to me to use the term "injury" in the context of the BART, maybe consider using "loss" or a similar term). Specifically, the BART-models mentioned above (Wallsten et al., 2005) were specifically developed to account for (subjective) explosion probabilities (e.g., to compare to the objective explosion probabilities). Moreover, in more recent research, the impact of different [explosion] probability conditions was directly assessed with respective prompts and modeling (Schürmann et al., 2019; Steiner et al., 2020).*

We have now changed all references to “injury” on the BART to “loss.” As stated above, we now incorporate these models in our summary of the BART. We additionally reviewed the work of Schurmann and colleagues (2019) and associated BART models. We clarify that while these models involving loss probability exist, they are not considered in models that also include loss magnitude due to the nature of the task (i.e., the balloon either explodes or does not), a gap that APRT’s design fills (pp. 5-6).

7. *The authors argue (p. 7) that previous tasks may be constrained as they consist of games without "a real-world equivalent". In line with this argument, it has been proposed in the literature that some tasks (including the BART) may be constrained in terms of their*

*generalizability (see comment above) because they lack a representative design (Steiner et al., 2020). Thus, it would be important to introduce how / why the four settings implemented in the APRT are ecologically more relevant (e.g., encountering a tiger does not seem an overly realistic real-life experience).*

We have updated our explanation of APRT to further cement this point. We note that existing tasks are likely ecologically constrained due to poor representative design (p. 6), as noted by Steiner and Frey (2021). We therefore argue that APRT possesses relatively better representative design, though we concede that the scenarios presented in APRT are rare (but still realistic) occurrences of physically risky behavior, but also that the extreme nature of these allows for maximized effect sizes (p. 16).

8. *The authors claim that an advantage of the novel task is that a participant cannot simply infer the probability of a gain from the probability of a loss (or vice versa), as there are also "neutral" outcomes. Thus, can one argue that this is a three-outcome setting? If so, it would be interesting to relate that to the respective research (e.g., on monetary gambles) that has used setups other than the typical two-outcome gambles.*

Indeed, APRT’s status as a three-outcome task makes it distinct from other common risk taking tasks such as BART. We have updated our introduction (pp. 8-9) to review research of three-outcome gambling tasks, specifically the differing models used to understand the influence of loss and gain probabilities and magnitudes (i.e., prospect theory vs. cumulative prospect theory).

9. *Relatedly, I wondered what to make of the additional dimension of "health". It seemed to me as if the participants' had to deal with two non-fungible "currencies" in the task (i.e., maximize payoffs in terms of the points, or maximize health)? Can the authors elaborate further on this issue?*

We have updated our explanation of APRT to address this issue (pp. 16-17). We clarified the secondary nature of not only the health, but also injury count outcome. We note that participants are not instructed to maximize health or minimize injuries, in contrast to the points outcome, as they are explicitly instructed to maximize their score. Thus, it is made clear to participants that the number of points is the primary outcome in this task, which requires more frequent Go presses to earn.

10. *Finally, I was also somewhat puzzled by way the information about the probabilities of success / failure was communicated to participants (the colored / striped borders). The authors argue that this procedure "protects the task from learning effects", but I could not quite follow this rationale. In fact, this creates an additional learning component exactly *because* participants have to remember (and recall) the meaning of this symbolic representation (which in itself could lead to noisier behavior). Second and under the assumption that participants could perfectly recall the meaning of the various borders, how / why would this be any different from directly depicting the probabilities?*

We appreciate this excellent point and reflected on its implications considerably. We added an additional limitation regarding potential issues with how the probabilities of loss and gain on APRT are presented to participants on p. 46, paragraph 2. While the purpose of the border color/pattern scheme was to *prevent* learning effects, the non-explicit nature of depicting probabilities creates a potential learning effect, wherein some participants will correctly remember the border rules more often than others. We note that future studies should explore whether directly depicting the probabilities to participants does result in significant behavioral differences on the task compared to the current method. However, we also noted that our primary concern with possibly doing so is overwhelming participants with numerical information during the task.

11. *I found the theoretical motivation for the various hypotheses (p. 11) relatively thin. This is particularly an issue as the model implemented to test these hypotheses is very flexible (i.e., many terms and interactions; generally many exploratory analyses).*

On p. 10, paragraph 2, we now reiterate earlier citations to better justify our hypotheses. We also clarified that we expected injury-related variables to have larger effects due to the greater penalty of “losing” in physical risk taking, such as bodily injury. That said, we also clarified the largely exploratory nature of our hypotheses, noting that our analyses intentionally allowed the study of all possible main effects and two-way interactions. On p. 11, paragraph 1, we emphasize our awareness of the multiplicity of comparisons and that they were statistically corrected for in our analyses. Our hypotheses were thus the effects we thought were *most* important to examine and most in line with the extant risk-taking literature, rather than questions that were specifically meant to be answered by our methods.

12. *Relatedly, although participants completed a number of trials, I wondered whether the various parameters are fully identified in this model. A simulation / model recovery analysis could be informative and boost the confidence in the robustness of the results.*

The extent to which the parameters are fully identified in the APRT task has now been clarified through the addition of a new supplementary table, which depicts how often each condition of each APRT factor appeared across the 64 task trials (referenced on p. 16, paragraph 1). Additionally, while we appreciated the suggested implementation a simulation/model recovery analysis, we were unfamiliar with this technique and thus did not feel comfortable attempting to implement it in the current study. However, we will keep this suggestion in mind and potentially collaborate with a colleague knowledgeable with this technique in future studies.

13. *Schonberg, T., Fox, C. R., & Poldrack, R. A. (2011). Mind the gap: Bridging economic and naturalistic risk-taking with cognitive neuroscience. Trends in Cognitive Sciences, 15(1), 11–19. https://doi.org/10.1016/j.tics.2010.10.002*

*Schürmann, O., Frey, R., & Pleskac, T. J. (2019). Mapping risk perceptions in dynamic risk-taking environments. Journal of Behavioral Decision Making, 32, 94–105. https://doi.org/10.1002/bdm.2098*

*Steiner, M. D., & Frey, R. (2020). Representative Design in Psychological Assessment: A Case Study Using the Balloon Analogue Risk Task (BART). https://doi.org/10.31234/osf.io/dg4ks*

*Wallsten, T. S., Pleskac, T. J., & Lejuez, C. W. (2005). Modeling behavior in a clinically diagnostic sequential risk-taking task. Psychological Review, 112(4), 862–880. https://doi.org/10.1037/0033-295X.112.4.862*

*Yechiam, E., Busemeyer, J. R., Stout, J. C., & Bechara, A. (2005). Using cognitive models to map relations between neuropsychological disorders and human decision-making deficits. Psychological Science, 16(12), 973–978. https://doi.org/10.1111/j.1467-9280.2005.01646.x*

As can be seen in our previous responses, these references have been integrated throughout the manuscript to enrich our review of the literature and highlight previous operationalizations of the elements of risky decision making in existing tasks. Several new references have also been added throughout to further explicate the theoretical background surrounding risk taking and the generation of the APRT task.

Reviewer 2

1. *The manuscript “the assessment of physical risk taking: construct validation of a new behavioral measure” presents a new method of assessing physical risk taking which simultaneously varies probability and reward in more realistic contexts than existing behavioral measures. As an initial presentation, the paper is adequate in its rationale and uniqueness. The data presentation is fairly thorough and there is some initial evidence of construct validity. Therefore as an initial presentation I believe the manuscript is adequate, however I believe the limitations and future directions should be more explicit on what is not in the manuscript (and given the scope of the paper probably shouldn’t be in this paper). First, construct validity is not a conclusive state but a process of accumulation that would need to continue in future studies.*

The reviewer makes a critical point here, and throughout the limitations and future directions, we now highlight a number of the aspects of construct validity that would require further examination in future studies. Furthermore, we have changed the title of the manuscript to “The Assessment of Physical Risk Taking: Preliminary Construct Validation of a New Behavioral Measure” to better reflect the introductory nature of the present data.

2. *Second, criterion validity is absent and would be required before the measure could be used in any real-world applications.*

We agree that establishing criterion validity will be an important step in the development of APRT as a measure of physical risk taking with real-world implications. We address several such possible criteria on p. 47, paragraph 2.

3. *Third, a direct comparison between the APRT, BART, IGT, and other major behavioral risk-taking measures would be required to illustrate the practical advantages the APRT claims.*

The reviewer makes an excellent point here, as the incremental utility of many measures are insufficiently studied. We discuss this requirement on p. 47, paragraph 2.

4. *And finally, given all of these points, it should be made clear that the APRT requires further evidence of validity and utility prior to any applied use.*

We have updated our discussion and limitations (pp. 47-48) to clarify this need for further investigation of the APRT’s validity and utility before recommending applied uses of the task (though we still discuss what these applied uses might be). Specifically, we highlighted the need for further studies examining the criterion validity of APRT (e.g., future substance use, future automotive or adventuring accidents resulting in injury), as well as for studies directly comparing APRT performance to those on other behavioral tasks (e.g., BART, CCT, IGT).

5. *Fourth, the issue of non-response mentioned in the limitations needs further study with larger and more diverse samples.*

Acknowledging this point, we also noted the need to determine if the low response rate of some participants is present in larger, more diverse samples (pp. 45-46), or if it remains after the removal of the delay condition or alterations to some of the variables (e.g., shrinking the gap between the two reward magnitude conditions).

Again, we thank you for the opportunity to revise this manuscript and respond to the critiques. We intend to address the limitations of the current project in our own future studies, and we hope that our revisions have satisfactorily established the strong foundation the current evidence provides for APRT as the next evolution of behavioral risk taking assessment in psychological research.

Sincerely,

Stephen D. Benning

Associate Professor

Department of Psychology

University of Nevada, Las Vegas
