# Supplementary material for: The assessment of physical risk taking: Preliminary construct validation of a new behavioral measure
Source: PLoS One. 2021 Oct 28;16(10):e0258826. doi: 10.1371/journal.pone.0258826 (PMC8553120; doi:10.1371/journal.pone.0258826)
Supplement: Attachment — Submitted filename: Response to Reviewers.docx [file pone.0258826.s010.docx]

September 18, 2021

Dear Dr. Konstantinidis,

We very much appreciate the opportunity to continue to revise our manuscript on our new behavioral task, the Assessment of Physical Risk Taking. We have removed one citation in our revised manuscript, as this was determined to have been a preprint that ultimately was never published in a peer-reviewed journal, and two other articles cited alongside it still provided sufficient evidence for the point being made. Our other revisions have allowed us to add clarifications on the nature of our new task and future directions that would be helpful to pursue in its further validation. Below, we detail our responses to the expert reviewer’s comments.

1. *On p. 4 the authors argue that previous tasks do not correlate strongly with real-life behaviors and hence, that there is a need for more complex behavioral tasks. Alike, on p. 46 the authors argue that maybe even "more complex simulations" are required (i.e., to capture additional dimensions relevant to risk taking). So this is actually rather a comment than a suggestion, but personally I do not quite agree with this conclusion. In my view many tasks are already too complicated, often making it difficult to identify the exact reasons why people do (vs. do not) take risks in these tasks (especially if only few trials are collected; see also comment 3 below). An alternative and in my view more promising approach consists of actually developing simpler tasks, which may capture specific risk-related dimensions more precisely. Again, this is my personal opinion and should not prevent publication of this paper; the respective debate needs to be conducted in the field.*

We appreciate the transparency in the reviewer’s opinion, and we reflected on the pros and cons of more complex experimental designs, both before and after submitting this manuscript for peer review. We have expanded on this tension between parsimony and complexity in our discussion section with a new paragraph (middle of p. 47), noting that maintaining the complex design of APRT is contingent on providing further evidence for the robustness and magnitude of observed interaction effects, as well as their impact on real-world behavior.

2. *The study on the novel task aims to achieve many things at once (i.e., multiple conditions and various outcomes are compared against multiple measures and scales), and ultimately the reader may wonder how robust the reported effects indeed are. I appreciate that the authors acknowledged the exploratory nature of their research in the revision and that they sharpened their predictions where applicable. In my view, however, it could be helpful to make the exploratory nature even more explicit, in order not to raise any wrong expectations. For example, adding something like "An exploratory investigation of..." to the title and / or abstract may help setting expectations straight.*

We understand the importance of properly managing reader expectations and thus have updated the wording of the abstract to reflect the exploratory nature of aspects of the study. However, we did not make further edits to the title given that the current title already includes “preliminary” and that we preregistered our data analytic plan, so the study was not completely exploratory. Nevertheless, because we had relatively few specific hypotheses for how the APRT would perform, we corrected for multiple comparisons in both the GEE and correlational analyses across all the effects in each type of analysis, so we do not wish to undersell the value of the observed effects.

3. *With comments #1 and #2 in mind, I would find it essential that future research clarifies the identifiability and reliability of the parameters estimated in this task. Again, many things are being manipulated and measured here – but are the various dimensions indeed clearly separable, and is the number of trials sufficient to identify reliable response patterns? As outlined in my previous review, a model recovery analysis could be very instrumental to this end. Briefly, the idea is to implement various data-generating processes (e.g. simulate groups of people who take risks for different reasons in different conditions), to then estimate the models and evaluate whether the implemented effects can be recovered (or how strong these effects would need to be in order to be recovered reliably). The authors responded that they are not confident implementing such an analysis - fair enough, but it would be very helpful to have at least an explicit discussion of the option to run a parameter recovery study in the future, to thus clarify the reliability of the extracted parameters / effects.*

We appreciate the elaboration on this analytic option. We agree that the number of trials currently in APRT is a significant limitation on examining reliability of effects, but we note that adding additional trials would greatly increase risk of participant fatigue effects, were it even viable (i.e., if a sufficient number of pictures could even be obtained to create enough additional trials). Thus, we discuss implementing model recovery analyses in future studies (paragraph spanning pp. 46-47) to confirm the reliability of effects, as it is a more viable solution than increasing the number of trials within APRT to parametrically vary trial count to examine how many are required in each cell to optimize scores’ reliabilities.

4. *Finally and also with expectation management in mind, I wondered whether it would not be more accurate / informative to use "simulation of" instead of "assessment of" physical risk taking in the name of the task. The latter implies that some concrete real-life behaviors are assessed, which is naturally not quite the case as the authors also acknowledge in the paper.*

We now more concretely state in the abstract that the APRT simulates physically risky activities. We also added a sentence to the end of the abstract noting that further study is needed to establish the validity of APRT in assessing real-world physical risk taking. However, we did not change the title as suggested because it is indeed our goal to use APRT as an assessment of physical risk taking. Though it is true that the task itself uses simulated risk behaviors, the intention behind the measure was to provide a relatively minimally abstracted task for respondents to demonstrate their physical risk taking propensity. The term “assessment” implies a quantitative treatment of behavior that emphasizes the scores from the task, whereas using the term “simulation” tends to emphasize the qualities of the simulation itself. Thus, we believed retaining the APRT title was appropriate after disclosing the simulated nature of the task in the abstract and main text. In particular, given the popular connotation of “simulations” as being complex, realistic, and lifelike (e.g., virtual reality, media such as *The Matrix*), changing the name to “simulation of” may further mislead expectations, given the relatively simplistic nature of the task.

Again, we thank you for the opportunity to revise this manuscript and respond to the critiques. We reassert our intentions to address the limitations of the current project in our own future studies, and we hope that our revisions have satisfactorily established the foundation the current evidence provides for APRT as the next evolution of behavioral risk taking assessment in psychological research.

Sincerely,

Stephen D. Benning

Associate Professor

Department of Psychology

University of Nevada, Las Vegas
